# Calcipotriol/Betamethasone Dipropionate for the Treatment of Psoriasis: Mechanism of Action and Evidence of Efficacy and Safety versus Topical Corticosteroids

**DOI:** 10.3390/jcm13154484

**Published:** 2024-07-31

**Authors:** Paolo Gisondi, Tamara Gracia-Cazaña, Hjalmar Kurzen, Jordi Galván

**Affiliations:** 1 Section of Dermatology and Venereology, Department of Medicine, University of Verona, 37126 Verona, Italy; 2 Department of Dermatology, Hospital Miguel Servet, 50009 Zaragoza, Spain; tgraciac@salud.aragon.es; 3 Haut- und Laserzentrum Freising, 85354 Freising, Germany; hjalmar.kurzen@icloud.com; 4 Klinik und Poliklinik für Dermatologie und Allergologie am Biederstein, Technische Universität München, 80802 Munich, Germany; 5 Global Medical Affairs Department, Almirall S.A., 08022 Barcelona, Spain; jordi.galvan@almirall.com

**Keywords:** psoriasis, calcipotriol, betamethasone, topical corticosteroids, mechanism of action, efficacy, safety

## Abstract

The combined treatment with calcipotriol (Cal) and betamethasone dipropionate (BDP) has emerged as the leading anti-psoriatic topical treatment. Fixed-dose Cal/BDP is available in different formulations, including ointment, gel, foam, and cream. This review examines the mechanism of action of Cal/BDP underlying its therapeutic effect and compiles the evidence regarding its efficacy and safety compared to monotherapy with topical corticosteroids. The dual-action of Cal/BDP targets the inflammatory pathways and abnormal keratinocyte proliferation, both of them fundamental mechanisms of psoriasis pathogenesis. A large number of randomized, double-blind studies support Cal/BDP superiority over topical corticosteroids, demonstrating its broad efficacy across several degrees of psoriasis severity and its capability to provide early significant clinical improvements. This increased efficacy is achieved without negative effects on the safety profile, since the incidence of adverse effects reported with Cal/BDP is usually similar to that of BDP and even lower than that of Cal alone. The combination therapy rapid onset of action, coupled with a simplified dosing regimen, has been identified as crucial for improving long-term adherence and patient outcomes. In conclusion, Cal/BDP is confirmed as a versatile, effective, and convenient option for the patient in psoriasis management.

## 1. Introduction

Psoriasis is a chronic, inflammatory dermatologic disorder characterized by accelerated epidermal cell proliferation that culminates in the formation of erythematous plaques with silvery-white scaling [1]. Affecting 1% to 3% of the Western population, this condition predominantly presents as plaque psoriasis in 80–90% of the cases [2]. Notably, a significant proportion of psoriasis (75%) is classified as mild, involving less than 3–5% of the body surface area (BSA), and is typically responsive to topical therapeutic interventions [3].

The etiology of psoriasis is rooted in an aberrant immune response, with T-cells playing a central role [4]. In psoriasis, these T-cells exhibit hyperactivity, leading to an escalated proliferation of epidermal cells and the hallmark manifestations of the condition [5]. The interleukin (IL) 23/helper T (Th) type 17 axis represents the central immune pathway. In psoriasis, an increased production of IL-23 and IL-17 produces a self-amplifying inflammatory response in keratinocytes, leading to epidermal hyperplasia, epidermal cell proliferation, and the recruitment of leukocyte subsets into the skin [4]. Genetic predisposition contributes significantly to the susceptibility to psoriasis [6]. Additionally, environmental factors such as stress, infections, and certain medications can exacerbate the symptoms [7]. A comprehensive understanding of its pathophysiology is imperative for devising effective treatment strategies.

The management of psoriasis is multifaceted, aiming to mitigate symptoms, reduce inflammation, and improve patient’s quality of life [8]. The therapeutic arsenal includes topical treatments, phototherapy, systemic medications, and biologic agents. For mild-to-moderate conditions, first-line treatments often encompass topical corticosteroids, vitamin D analogs, and retinoids to control inflammation and scale formation. Phototherapy, utilizing ultraviolet B (UVB) or psoralen plus ultraviolet A (PUVA), is effective for more extensive skin involvement. Severe cases need systemic treatments like methotrexate, cyclosporine, and dimethyl fumarate. In recent years, the introduction of biologic therapies targeting specific disease pathways, such as tumor necrosis factor α (TNF-α) and IL-17 and IL-23 inhibitors, represents a significant progress in the treatment of moderate-to-severe psoriasis [1,9].

Topical treatments play a predominant role in the management of psoriasis, either as monotherapies or in conjunction with systemic interventions in cases of extensive disease (involving >10% BSA) [10]. Corticosteroids have been a mainstay in topical psoriasis management [11]. However, concerns over long-term side effects have spurred the exploration of alternatives [11]. Topical vitamin D analogs are a cornerstone in the management of mild-to-moderate psoriasis due to their efficacy and favorable safety profile with minimal side effects over both short- and long-term use [12]. 

Treatment with calcipotriol/betamethasone dipropionate (Cal/BDP) combines a vitamin D analog with a corticosteroid, offering a synergistic approach. This dual-action mechanism, with Cal addressing keratinocyte turnover and differentiation, and BDP targeting inflammation, has led to the approval of various Cal/BDP formulations for mild-to-moderate psoriasis [13]. Clinical guidelines highlight the efficacy and safety of Cal/BDP, positioning it as a balanced combination, providing efficacy but with minimized corticosteroid-related side effects. The choice between Cal/BDP and corticosteroid therapy remains patient-specific, influenced by psoriasis severity, lesions’ location, and patient preferences [14,15]. This review intends to synthesize the current evidence on Cal/BDP combination therapy compared to topical corticosteroid monotherapy in psoriasis treatment. This information will help clinicians to understand the differences between the two therapies, allowing them to make better-informed decisions when choosing the most suitable treatment for each patient.

## 2. Methods

This narrative review summarizes the published scientific evidence regarding the mechanism of action of Cal and corticosteroids in the treatment of psoriasis. It also reviews the results of clinical studies that compare the Cal/BDP combination with any topical corticosteroid in the treatment of psoriasis.

Published studies that evaluated the mechanism of action of Cal and corticosteroids (mainly BDP), either as combined therapy or as monotherapy, in the treatment of psoriasis were first identified through a broad search conducted in the scientific database PubMed. Original articles published after 2000 in English were searched.

Next, clinical studies published comparing the Cal/BDP combination with any topical corticosteroid in the treatment of psoriasis were identified through a broad search of the scientific literature, including international databases such as PubMed, Scopus, ScienceDirect, and Google Scholar. Accessible book abstracts from main dermatology meetings and congresses (e.g., the World Congress of Dermatology and those organized by the European Academy of Dermatology and Venereology, the American Academy of Dermatology, the Skin Inflammation and Psoriasis International Network, the British Association of Dermatologists, and the European Society for Dermatological Research) were also included. Literature published in English was searched. Original articles, abstracts, and meta-analyses published after 2000 were included.

## 3. Mechanism of Action of Cal/BDP and Its Individual Components

**Figure 1:**
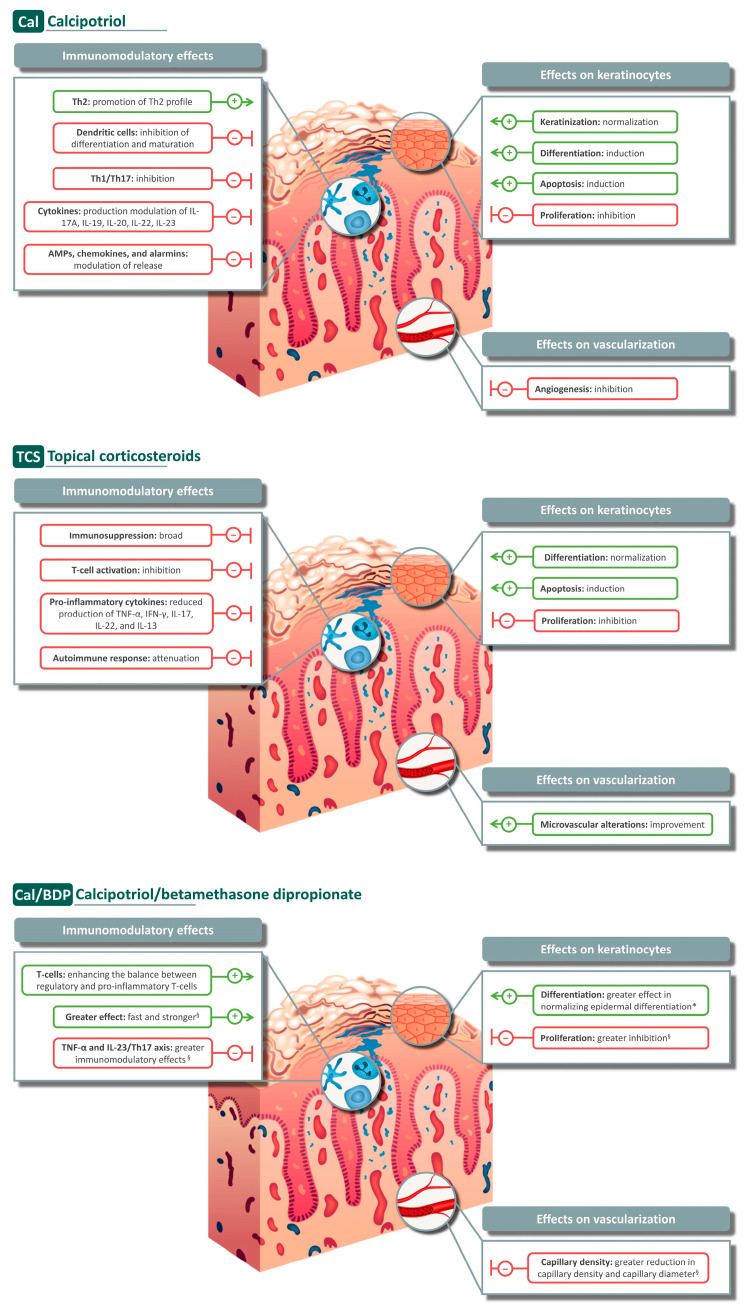
Summary of the main effects exerted by calcipotriol, corticosteroids, and calcipotriol/betamethasone dipropionate on immune cells, keratinocytes, and vascularization. * compared to Cal or clobetasol; ^§^ compared to Cal and/or BDP. AMP: antimicrobial peptide, BDP: betamethasone dipropionate, Cal: calcipotriol, IFN-γ: interferon-γ, IL: interleukin, Th: helper T-cell, TCS: topical corticosteroids, TNF-α: tumor necrosis factor α. Source: Figure created by the authors based on the content of Section 3.1, Section 3.2 and Section 3.3.

The mechanism of action of Cal and topical corticosteroids, alone or in combination, has been extensively studied through *in vitro* models, murine models, and clinical studies in the field of psoriasis. These agents exert multiple effects on the immune system, keratinocytes, and vascularization, as will be described in the following subsections (Figure 1).

### 3.1. Mechanism of Action of Calcipotriol (Cal)

One of the most important aspects of Cal mechanism of action lies in its immunomodulatory and anti-inflammatory activity. *In vivo* studies in murine models and clinical trials have demonstrated that Cal can reduce swelling, hyperplasia, and inflammatory infiltration [16,17,18]. Cal exerts its immunomodulatory effects by acting on specific populations of immune cells and cytokines. In particular, Cal hinders the differentiation and maturation of dendritic cells [19] and promotes a Th2 profile while suppressing Th1/Th17 cells [19,20], a highly relevant finding considering the pivotal role played by the IL-17/IL-23 axis in psoriasis pathogenesis [21]. Cal inhibits Th17 cells and IL-17-producing CD8+ T-cells and reduces the production of several cytokines, including IL-17A, IL-22, IL-23, and IFN-α [17,22]. It also modulates immune cell infiltration, notably neutrophils, Langerhans cells, and γδ T-cells, which are crucial IL-17 producers in the skin and are associated with psoriatic inflammation recurrence [16,17,22]. Cal action on psoriasis may also involve reduced production of IL-19 and IL-20 in keratinocytes, correlating with clinical and histological improvements [23].

Several studies, involving both *in vitro* models and clinical trials, also support the finding that Cal reduces cutaneous inflammation in psoriasis by modulating the release of key chemokines and antimicrobial peptides (e.g., human β-defensin, cathelicidin, psoriasin, koebnerisin), disrupting the pro-inflammatory ‘alarmin’-mediated loop, thereby inhibiting the disease inflammatory cascade [24,25,26,27,28,29,30].

Clinical trials and *in vitro* studies have also shown that Cal inhibits keratinocyte proliferation [30,31,32], normalizes their differentiation, induces their apoptosis [33,34], and promotes the normalization of keratinization [35]. Cal affects the expression of various keratins and markers including involucrin and transglutaminase [32] and it also directly inhibits the expression of IL-36 α/γ in keratinocytes, reducing skin inflammation [22]. In summary, Cal promotes the differentiation of keratinocytes towards a more normal phenotype, inhibiting the onset of psoriasis disease.

There is also evidence that Cal acts by modulating the expression of pro- and anti-angiogenic factors. In a transgenic murine model of psoriasis, Cal exerted an anti-angiogenic effect in psoriasis, downregulating vascular endothelial growth factor (VEGF) expression and upregulating pigment epithelium-derived factor (PEDF) expression. It also reduced microvascular density, all of which supports its role in attenuating the vascular hyperplasia often seen in psoriatic lesions [36].

### 3.2. Mechanism of Action of Topical Corticosteroids

It is well known that topical corticosteroids exert their therapeutic action in psoriasis through the suppression of the immune system and by inhibiting T-cell activation [19]. For instance, research on BDP in murine models of skin inflammation and psoriasis revealed its ability to inhibit skin inflammation, redness, erythema, thickening, and scaling [20,22,37]. BDP reduces the expression of Th1/Th17-related cytokines (i.e., TNF-α, IFN-γ, IL-17, IL-22, and IL-13) [20,22] and antimicrobial peptides (e.g., S100 calcium-binding proteins A8 and A9) [20]. Moreover, BDP attenuates the autoimmune response in psoriasis, lessening the infiltration and proliferation of leukocytes, CD4+ T-cells, Th17 cells, as well as altering the localization and proliferation of γδ T-cells [37]. These immunomodulatory effects contribute to mitigating inflammation in psoriasis.

Corticosteroids like betamethasone valerate, methylprednisolone aceponate, and BDP reduce keratinocyte proliferation and induce their apoptosis [37,38,39]. Additionally, treatment with betamethasone valerate leads to the normalization of epidermal protein expression, particularly in the suprabasal compartment of the epidermis [38]. These findings support the role of corticosteroids in normalizing epidermal proliferation and differentiation, which is crucial for treating psoriatic lesions [38,39].

Additionally, a favorable impact of corticosteroids on cutaneous microvascular dynamics has also been proposed. This observation is based on a clinical study involving psoriasis patients, where it was found that mometasone furoate attenuated clinical and capillaroscopic alterations in psoriatic plaques [40].

### 3.3. Mechanism of Action of Cal/BDP

The mechanism of action of Cal/BDP involves complementary and additive effects of the individual components on immune cells. Although both Cal and BDP have immunomodulatory effects, Cal specifically targets Th1/Th17 cells, promoting a Th2/Treg profile [41,42], whereas BDP exerts a broader inhibitory effect, acting on Th17 but also other T-cell subsets [42]. In addition, the combination of Cal and BDP surpasses the immunomodulatory action of either agent alone, enhancing the regulatory and pro-inflammatory T-cell balance, and exhibiting rapid and potent effects attributed to the synergistic interaction of the corticosteroid and Cal [41,42,43,44,45,46]. This synergy results in a more pronounced modulation of TNF-α and the IL-23/Th17 axis [41,47]. The Cal/BDP combination not only improves the balance between regulatory and pro-inflammatory T-cells [46] but also achieves more rapid and pronounced effects, attributable to the corticosteroid component [48]. This synergistic effect is particularly evident in the context of lymphocyte proliferation [49], where the combination of Cal and BDP increases the suppressive effect on cell proliferation compared to BDP alone.

Regarding its effects on epidermal populations, Cal/BDP produces marked improvements in clinical scores and reduces the levels of various biomarkers related to epidermal proliferation, differentiation, and inflammation [47,50,51,52,53]. The combination of Cal/BDP might exhibit a more pronounced impact on normalizing epidermal differentiation and in suppressing keratinocyte proliferation compared to either Cal [51,52], clobetasol [47,53], or BDP individually [53].

Cal/BDP has shown a greater improvement in microvascular changes [54,55,56,57,58,59], reducing the diameter and density of bushy capillaries more effectively than monotherapies [57]. Similarly, additional studies showed that Cal/BDP foam reduced the vascularization and stiffness of responsive psoriatic plaques and decreased capillary density, indicating a reduction in inflammation, normalization of epithelial keratinization, and improvement in angiogenesis [54,59]. 

In summary, when used together, Cal and BDP address multiple aspects of psoriasis in a complementary and synergistic manner.

## 4. Efficacy of Cal/BDP versus Topical Corticosteroids

The available fixed-dose Cal/BDP (Cal 0.005% and BDP 0.064%) treatment options in the European Union (EU) include different formulations such as ointment, gel, foam, and cream, all of which have demonstrated their efficacy treating psoriasis in clinical trials [60,61,62,63,64,65,66,67,68,69,70,71,72,73,74,75,76,77,78,79]. 

Two Cochrane systematic reviews provide a comparison of the efficacy of different topical treatments in chronic plaque psoriasis of the body [80] and scalp [80,81]. The comprehensive review conducted by Mason et al. included 177 randomized controlled trials [80]. In body psoriasis, a combination therapy (vitamin D analogs plus corticosteroid) was more efficacious than either individual product used as monotherapy. Cal combined with BDP was more efficacious than Cal alone and also more efficacious than BDP alone. These changes equate to approximately 0.6 and 0.4 points, respectively, on a six-point Investigator Assessment of Global Improvement (IAGI) scale. When focused on scalp psoriasis, both reviews concluded that the combination therapy and the corticosteroid monotherapy exhibited greater efficacy compared to monotherapy with vitamin D [80,81].

To further address the efficacy of the different formulations of Cal/BDP and topical corticosteroids in the treatment of psoriasis, numerous clinical trials have directly com-pared Cal/BDP ointment, gel, and foam versus (vs.) topical corticosteroids. However, to our knowledge, there are still no published studies that have compared the Cal/BDP cream with corticosteroids. The results obtained in these studies are summarized in Table 1 and will be reviewed in the following subsections.

### 4.1. Cal/BDP Ointment

Several randomized, double-blind studies have consistently demonstrated the superior efficacy and a more rapid onset of action of Cal/BDP ointment, applied either once or twice daily, compared to its individual components (Cal and BDP) or vehicle in treating psoriasis vulgaris [61,62,63,64]. Interestingly, even a once-daily Cal/BDP regimen achieved higher efficacy than BDP applied twice daily [64]. Cal/BDP achieved a greater decrease in the Psoriasis Area Severity Index (PASI) score, with a faster onset of action and significant improvements being apparent as early as week 1 [61]. These improvements extended up to 4 [64] or 12 weeks [61,62,63], compared to the components alone or the vehicle. This enhanced efficacy was notable across various severities of the disease, with a higher proportion of patients achieving significant PASI reduction with the combination than with the Cal and BDP monotherapies [61,62,63,64]. 

Apart from large-scale clinical trials, smaller studies have investigated specific aspects of the effectiveness of Cal/BDP ointment. One such study, for example, examined the clinical effectiveness and the impact of Cal/BDP, as well as of its individual components, on microcirculatory changes in patients with psoriasis. Cal/BDP not only offered better clinical improvement than both monotherapies, but also facilitated more efficacious microvascular restoration, leading to improvements in dermatological signs such as erythema, infiltration, and desquamation [57]. 

Several clinical trials have also evaluated the efficacy of Cal/BDP ointment in comparison to corticosteroids other than BDP, yielding insightful results about its relative efficacy. One such study showed that the efficacy of clobetasol propionate spray was superior to that of Cal/BDP ointment, with a higher proportion of patients achieving a rating of clear or almost clear disease according to Overall Disease Severity [65,66]. In another non-inferiority study, betamethasone valerate dressing was non-inferior to Cal/BDP ointment [67]. Furthermore, Cal/BDP ointment was compared with a 0.1% dexamethasone cream in a separate trial. This comparison revealed that Cal/BDP was more efficacious in reducing the total score of itching, erythema, infiltration, and scales in target lesions than the dexamethasone cream, suggesting a superior performance of Cal/BDP ointment in alleviating these specific symptoms and signs associated with psoriasis [68].

Data from individual clinical trials were synthesized in several pooled analyses and meta-analyses. These analyses confirm the findings reported in individual trials, positioning Cal/BDP ointment as a highly efficacious treatment option for psoriasis, superior to the monotherapy components [82,83,84,85,90].

### 4.2. Cal/BDP Gel

Numerous clinical studies have thoroughly assessed the efficacy of Cal/BDP gel against other corticosteroids in treating different forms of psoriasis, offering a broad perspective on its therapeutic efficacy.

Two multicenter, prospective, randomized clinical trials compared the efficacy of Cal/BDP gel to that of the respective monotherapies in treating body psoriasis [72,73]. One of these trials revealed that at week 4, the proportion of responders (i.e., patients whose disease was clear or very mild and who had at least a two-step improvement in the Investigator’s Global Assessment [IGA]) in the Cal/BDP group was comparable to that in the BDP group and notably higher than those in the Cal or vehicle groups. By week 8, a significantly higher percentage of participants in the Cal/BPD group had achieved disease control compared to the three other treatment groups, i.e., Cal, BDP, and vehicle. Additionally, a *post-hoc* analysis specifically focusing on patients with mild-to-moderate disease underscored the superior efficacy of Cal/BDP over comparative treatments at both 4- and 8-week evaluation points [72]. In the other study, a four-arm trial, the proportion of subjects achieving a controlled disease at week 4 was higher in the Cal/BDP group compared to the groups treated with Cal and vehicle but not to that treated with BDP. However, by week 8, Cal/BDP had exceeded the efficacy of all other treatments. Moreover, patients treated with Cal/BDP also exhibited greater improvements in reported quality of life compared to those receiving either vehicle or BDP [73].

One study used data from a U.K. general practice database to examine the time until subsequent disease worsening or the need for secondary care referral following different topical therapies in patients with mild-to-moderate psoriasis. It is to note that referral to secondary care is recommended when psoriasis remains unresponsive to topical therapy. The study concluded that patients treated with Cal/BDP gel were less likely to require secondary care or experience disease worsening compared to those receiving other treatments (Cal/BDP ointment or topical corticosteroids only) [91]. 

In a trio of multicenter, prospective, randomized clinical trials, the efficacy of a combined therapy using a Cal/BDP gel formulation was meticulously assessed in scalp psoriasis compared to that of its active ingredients individually or of the vehicle [69,70,71]. The findings of these studies consistently suggest that the Cal/BDP combination therapy not only showed a favorable tolerability profile but also showed a better efficacy profile compared to monotherapies involving either Cal [69,71] or BDP [69,70,71]. The trials used different evaluation criteria, including the IGA [69,71] and the total sign score [70], which quantified the severity of key psoriasis signs such as redness, thickness, and scaliness. A notable aspect of the Cal/BDP regimen was its more rapid onset of action when compared to the individual monotherapies [69]. According to the IGA, 57.5% of the patients treated with Cal/BDP achieved ‘absent’ or ’very mild’ disease at week 2, a percentage only surpassed by Cal treatment at week 8 (64.0%) and not even reached by BDP (36.8%) or the vehicle (22.8%) at this last time point. The combination therapy demonstrated significantly greater efficacy than BPD in the reduction of the total sign score after two weeks and at 8 weeks [70]. This early onset of action further underscores the therapeutic advantage of the combined Cal/BDP gel treatment in managing scalp psoriasis.

Additionally, another study compared a non-alcoholic mometasone furoate emulsion with Cal/BDP treatment in patients with non-severe scalp psoriasis. The non-alcoholic mometasone emulsion achieved greater acceptability to patients and physicians than the Cal/BDP gel for the treatment of scalp psoriasis; however, both topical treatments were similarly effective in terms of disease severity and quality of life [86]. 

The therapeutic efficacy of Cal/BDP gel in comparison to other topical treatments for scalp psoriasis was also investigated through a series of pooled analyses and a meta-analysis incorporating data from individual clinical trials. One pooled analysis concluded that Cal/BDP gel not only was more efficacious, but also had a faster onset of action compared to its individual components in scalp psoriasis [87]. Additionally, a systematic review and meta-analysis on scalp psoriasis compared the efficacy of Cal/BDP gel against other commonly used topical treatments for this condition, including BDP and betamethasone valerate. The results showed that Cal/BDP gel was more efficacious than the standard topical therapies, including corticosteroids, vitamin D analogs, shampoos formulated with coal tar, and shampoos combining coal tar with vitamin D analogs [88]. 

### 4.3. Cal/BDP Foam

Several studies compared the efficacy of Cal/BDP foam with that of other topical treatments for both body and scalp psoriasis. 

A study focusing on body and scalp psoriasis established that Cal/BDP foam was more efficacious than monotherapy with Cal or BDP in achieving treatment success for body psoriasis, as determined by the Physician Global Assessment (PGA) scoring system. In the treatment of scalp psoriasis, Cal/BDP foam was found to be as efficacious as BDP [74,75] and superior to Cal [76,77]. In the context of body psoriasis, an additional study compared the effects of Cal/BDP foam with those of other treatments, including Cal/BDP ointment, BDP foam, and vehicle. Cal/BDP foam demonstrated a significant improvement in anti-psoriatic effect over Cal/BDP ointment, BDP foam, and vehicle foam alone, as evidenced by a greater reduction in the total clinical score, which encompassed the sum of the scores for erythema, scaling, and lesional thickness.

Several clinical trials have also compared the efficacy of Cal/BDP foam with that of other corticosteroids. One study revealed that Cal/BDP foam not only displayed superior efficacy but also demonstrated a faster onset of action compared to betamethasone valerate-medicated plasters, even in difficult-to-treat areas [79]. Another study compared non-invasive clinical and microscopic features in a psoriatic target lesion treated with Cal/BDP foam or clobetasol propionate cream. The results concluded that Cal/BDP foam was more efficacious than clobetasol, obtained better patient satisfaction, and induced greater reduction in hyperkeratosis/acanthosis, irrespective of the baseline level of epidermal hyperplasia [60]. 

The efficacy and safety of Cal/BDP foam have been further investigated through the aggregation of data from individual clinical trials. A pooled analysis of data from three phase II/III studies further substantiated the efficacy of Cal/BDP foam across all assessed body areas, irrespective of baseline disease severity. The review concluded that Cal/BDP foam achieved higher treatment success and a greater reduction in modified PASI (mPASI), and a higher proportion of patients reaching PASI 75 by week 4, compared to the other treatments (Cal/BDP ointment, Cal foam, BDP foam, or vehicle foam) [92].

## 5. Safety

The safety of topical treatments is a critical consideration in selecting appropriate therapies for psoriasis [93]. Examining the individual components of Cal/BDP, the current knowledge indicates that vitamin D analogues are primarily associated with perilesional skin irritation, which is attributed to increased local blood flow [19]. In contrast, topical corticosteroids, when used over an extended period, present the risk of disease rebound, characterized by a worsening of the condition following the discontinuation of treatment, skin atrophy manifesting as thinning of the skin, and tachyphylaxis, which refers to a diminished response to the drug over time [80,94]. Additionally, a transient reduction in the hypothalamic–pituitary–adrenal (HPA) axis function, evidenced by decreased cortisol plasma levels, has been observed in up to 48% of patients treated with topical corticosteroids. However, this reduction is typically reversible within a few weeks and is not commonly associated with clinical symptoms, even during a prolonged maintenance treatment [94]. It is important to note that cases of prolonged HPA axis suppression are predominantly linked to the misuse of topical corticosteroids, specifically their extended daily application over several years and across large body surface areas [94].

Clinical trials, safety analyses, and systematic reviews comparing Cal/BDP ointment, gel, and foam formulations vs. topical corticosteroids have collectively demonstrated a favorable safety profile for treating psoriasis. Across these studies, Cal/BDP ointment and gel were consistently well tolerated, with adverse event (AE) rates comparable to those of BDP and lower than those associated with Cal monotherapy [61,62,63,64,69,70,71,72,73,83,88,90,95,96]. The foam formulation maintains a safety profile analogous to that of its individual constituents in the treatment of scalp psoriasis [73,74,97]. 

Most AEs reported in clinical trials with Cal/BDP application were mild, and the most common were nasopharyngitis, application site pain, skin irritation, burning sensation, pruritus, and upper respiratory tract infection [61,62,63,64,69,70,71,72,73,74,75,83,88,90,95,96,97].

Considering that HPA suppression may occur if a potent corticosteroid is absorbed to a significant extent [94], two prospective, randomized, active-controlled, double-blind trials, both short- and long-term, investigated the effect of Cal/BDP ointment on HPA axis function. The results, spanning from 4 to 52 weeks, reported no instances of adrenal suppression [98]. Additionally, an open, non-controlled 8-week trial involving patients with extensive psoriasis covering 15–30% of the BSA treated with Cal/BDP gel found that 4.7% of the subjects exhibited signs of HPA at week 4. The authors concluded that HPA suppression may occur only in a small subset of patients with extensive psoriasis when treated with large volumes of topical Cal/BDP gel [99].

The safety profile of Cal/BDP ointment was also explored through various comparative studies with other corticosteroids distinct from BDP. A multicenter randomized study that compared Cal/BDP ointment and clobetasol propionate spray revealed a higher incidence of stinging or burning sensations in the clobetasol-treated group, suggesting a trade-off between efficacy and tolerability for this corticosteroid [65,66]. Subsequently, a larger phase IV trial that evaluated Cal/BDP ointment and betamethasone valerate dressing showed similar rates of AEs in the two groups [67].

Regarding the Cal/BDP cream based on the polyaphron dispersion (PAD) technology (Cal/BDP PAD-cream), all adverse reactions observed in clinical trials had a frequency below 1% (uncommon). The most common System Organ Class was ‘General disorders and administration site conditions’ (2.7%), with application site pain, irritation, and pruritus being the most frequent local site reactions (each with a frequency of 0.7%) [100]. The adverse reactions reported for Cal/BDP PAD-cream were similar to those of other Cal/BDP formulations [101].

## 6. Comparison of Cal/BDP Formulations

Previous research has consistently shown that Cal/BDP aerosol foam formulation outperforms its ointment and gel predecessors in managing plaque psoriasis [102,103,104]; this is possibly due to the foam’s propellant potential and its ability to create stable supersaturated solutions, enhancing skin penetration and improving active ingredients’ bioavailability [105].

While patients often prefer gels or foams for their ease of application compared to ointments [106], ointments may be most effective for nail psoriasis, and gels are preferred by those with scalp psoriasis [107].

Recently, a new Cal/BDP combination formulated in cream has joined the topical armamentarium for the treatment of plaque psoriasis. This novel cream formulation based on PAD technology has showed superior treatment success rates compared to the gel formulation [100,108]. A pooled analysis from two phase 3 trials demonstrated that Cal/DBP PAD-cream achieved better efficacy and patient-reported outcomes. For most efficacy variables (PGA, Subject Global Assessment [SGA], mPASI improvement, PASI 75, Dermatology Life Quality Index [DLQI] improvement), Cal/BDP PAD-cream achieved a statistically significant difference with respect to Cal/BDP gel from week 4 onwards, and for some variables (itch, DLQI satisfaction), this difference could already be seen after one week of treatment [100,101,108]. 

That is, both foam and PAD-cream Cal/BDP formulations have been shown to be superior to the Cal/BDP gel formulation. As no head-to-head data are available, two matching-adjusted indirect comparison (MAIC) approaches were done to compare Cal/BDP foam vs. Cal/BDP-PAD cream. 

In line with the NICE recommendation [109], which stipulates that only ‘anchored’ forms of population adjustment should be used when a common comparator is available, the presence of Cal/BDP gel in studies conducted with both Cal/BDP PAD-cream and Cal/BDP foam formulations allowed for the undertaking of an anchored MAIC [110,111]. 

The comparative anchored analysis revealed no statistically significant differences in PGA success, mPASI75, or DLQI outcomes between Cal/BDP PAD-cream and Cal/BDP foam when assessed after their respective recommended treatment durations (8 weeks for the cream and 4 weeks for the foam). However, regarding treatment satisfaction after one week, Cal/BDP PAD-cream yielded significantly better results than Cal/BDP foam across all questionnaire domains except for ’easily incorporated into daily routine’ [110].

The anchored comparisons in a second MAIC corroborated the efficacy findings when comparing Cal/BDP PAD-cream and Cal/BDP foam in terms of PGA success and mean mPASI change from baseline at their recommended treatment durations for both formulations [111]. The consistency of the results observed in both anchored MAICs provides strength and reliability to the observed data.

The preference for Cal/BDP PAD-cream in terms of satisfaction and convenience was further validated in a published clinical trial involving 150 patients with scalp or body psoriasis, where the preference for Cal/BDP PAD-cream vs. the foam was evaluated. Cal/BDP PAD-cream surpassed Cal/BDP foam on various specific measures of satisfaction and in overall satisfaction assessments. Moreover, a recent survey showed that patients with plaque psoriasis treated with Cal/BDP PAD cream confirmed high treatment satisfaction and good adherence to it under real-world treatment conditions. They highlighted its ease/convenience of use, tolerability, rapid onset of action, and positive impact on their personal appearance and self-esteem [112,113].

## 7. Discussion

This review presented the synergistic mode of action of the combination of Cal and BDP, and its efficacy and safety in managing psoriasis.

The pathogenesis of psoriasis is underpinned by a complex interplay of immuno-logical and dermatological mechanisms, necessitating treatments that can address both inflammation and abnormal keratinocyte behavior [114]. Cal and BDP have additive and complementary pharmacological effects, enhancing the overall treatment efficacy [46]. The Cal/BDP combination leverages Cal’s ability to modulate keratinocyte proliferation and differentiation, alongside BDP’s potent anti-inflammatory and immunosuppressive effects [46,114]. This dual mechanism of action targets key inflammatory pathways, notably, TNF-α and the IL-23/Th17 axis [41,115], which are pivotal in driving the psoriatic process. Additionally, certain studies suggest that the Cal/BDP combination therapy reduces epidermal proliferation and normalizes keratinization more effectively than either monotherapy [19,51,53]. This combination synergistically modulates various T-cell subsets, positively influences systemic inflammatory markers, reduces tissue-resident memory T-cells (particularly in the epidermis), and normalizes skin microcirculation, which plays a crucial role in alleviating the inflammation and other underlying processes that drive the disease progression [116]. By addressing these aspects, the combination of these mechanisms significantly enhances the clinical efficacy of Cal/BDP, establishing it as a one of the main therapeutic options in psoriasis treatment [46,48,49]. 

Although the formulation of the treatment can have a major impact on its efficacy [78,89,107], the efficacy of Cal/BDP, irrespective of its formulation, has been reaffirmed through various randomized, double-blind studies. These investigations have not only confirmed the superior therapeutic outcomes of Cal/BDP over its individual components but have also showcased its efficacy across different severities of psoriasis. It particularly stands out in achieving more significant reductions in PASI compared to treatments with Cal and BDP alone [85]. Furthermore, this body of evidence highlights the treatment’s potential in delaying disease progression and reducing the need for referral to secondary care, while maintaining efficacy comparable to that of other established topical corticosteroids [86,91]. Specifically, in the treatment of scalp psoriasis, Cal/BDP stands out for its rapid onset of action and superior efficacy over other widely used topical therapies, contributing significantly to the growing body of evidence supporting its use [87,88]. Another significant finding is that, in comparison to the individual components, studies consistently show a quicker onset of therapeutic action with the combination, with significant clinical improvements within the first week of treatment [61,62,63,69,71,72,73,74,75], which is probably crucial for enhancing long-term adherence to treatment and improving overall outcomes. Despite the overall superior efficacy of Cal/BDP formulations, exceptions are noted. For instance, the efficacy of Cal/BDP ointment was found to be less than that of clobetasol propionate spray [65,66], suggesting that the therapeutic superiority of Cal/BDP might vary against very potent corticosteroids. This variability may reflect the nuanced interplay between drug potency, vehicle used in the formulation, and disease pathology [93], warranting further investigation into optimizing treatment strategies based on disease severity and patient-specific factors.

The vehicle characteristics are known to play a critical role in the effectiveness of topical therapy; they can alter the use/penetration of topical medications and hence the therapeutic effect [117], and additionally, patient’s preference for one vehicle over another is one of the most important factors affecting adherence, which in turn impacts its long-term effectiveness [101].

There are significant differences between the available formulations of Cal/BDP in the EU. As conventional Cal/BDP fixed combinations restricted to non-aqueous oil or paraffin-based formulations (e.g., ointment, gel, or foam) may be perceived as sticky or greasy by many patients [100], there was a need to develop a more patient-friendly topical treatment for psoriasis with the Cal/BDP combination. In this context, the novel cream formulation of Cal/BDP based on the PAD technology has emerged as a promising topical treatment for psoriasis. PAD formulations are oil-in-water dispersions with oil droplets encapsulated in a multi-molecular shell, protecting the active molecules from hydrolytic degradation and enhancing drug stability and the penetration of drugs like calcipotriene, betamethasone dipropionate, tacrolimus, and ciclosporin A. PAD formulations requires fewer surfactants, reducing skin irritation compared to conventional emulsions. Designed to be light, moisturizing, and quickly absorbed, PAD formulations increase patient satisfaction and adherence, particularly in chronic conditions like psoriasis and atopic dermatitis. This flexibility in drug design ensures efficacy, safety, and convenience [118], offering superior success rates compared to the gel formulation and overall patient acceptance [101] and satisfaction over Cal/BDP foam for the management of body and scalp psoriasis.

The safety of Cal/BDP for treating psoriasis is supported by several studies, elucidating its preferential status over monotherapies. The dual formulation tends to reduce the AEs commonly associated with the individual use of vitamin D analogs and corticosteroids. Specifically, vitamin D analogs are associated with skin irritation, but this is less frequent with Cal/BDP [19,61,62,63,113], due to the anti-inflammatory properties of BDP [97]. Corticosteroids, on their own, have a range of potential side effects, especially with long-term use, including the risk of disease rebound, skin thinning, and decreased drug effectiveness over time [80,94,119]. However, combining Cal with BDP appears to decrease the risk of such issues, particularly the risk of skin atrophy [55,56]. Additionally, comparative analyses indicate that Cal/BDP foam exhibits enhanced efficacy in preventing certain dermatological issues compared to clobetasol propionate cream, notably in the reduction of skin thickening and scaling [60]. Regarding the concerns about the HPA axis, which can be affected by corticosteroids, studies have shown that Cal/BDP does not induce significant perturbations in this endocrine axis, affirming its safety profile [94,98,99].

Although efficacy and safety are critical factors in the success of topical therapies for psoriasis, other variables such as skin type, plaque thickness, and, most notably, patient adherence, significantly influence the treatment outcomes [92]. In this regard, a simplification of the Cal/BDP treatment regimen through once-daily administration not only enhances convenience for individuals with psoriasis but also potentially improves treatment adherence. This is particularly relevant in the context of psoriasis management, where patient adherence to treatment regimens is a critical factor in achieving optimal clinical outcomes [120]. 

This article aimed to provide a comprehensive review of the Cal/BDP combination compared to monotherapy with topical corticosteroids. All identified articles were reviewed. Although the data presented here correspond to those in the last available publications related to this topic, some limitations may be inherent to the current type of this review, and the results should be interpreted within the context of a narrative review. One of the main limitations of narrative reviews is the lack of explicit criteria for publication selection. Despite using a clear search strategy to identify publications for this review, publication bias may be present, as journals tend to publish studies with positive or significant findings rather than negative or non-significant results [121]. Moreover, only publications written in English were analyzed for the purpose of this review.

## 8. Conclusions

The integration of Cal/BDP into a single combination therapy exhibits synergistic and complementary effects on the fundamental pathophysiology of psoriasis, leading to an enhanced therapeutic response. The Cal/BDP combination represents a forward step in the treatment of psoriasis. By synergistically modulating the immune response and epidermal changes characteristic of the disease, Cal/BDP offers an efficacious, safe, and patient-friendly option. Given its proven efficacy across various degrees of psoriasis severity and different locations, combined with a favorable safety profile and the convenience of a simplified dosing schedule, Cal/BDP is positioned as a cornerstone in the contemporary management of psoriasis. As the therapeutic landscape continues to evolve, the role of Cal/BDP in psoriasis care is increasingly recognized, marking it as a significant contribution to the field of dermatological therapy. Additionally, the selection of a suitable vehicle and consideration of patient preferences are crucial factors influencing treatment adherence and effectiveness.

## Figures and Tables

**Table 1:** Summary of studies of the efficacy of Cal/BDP formulations.

Cal/BDP Formulation and Dosage *	Comparator Dosage and Formulation ^†^	Treatment Duration	Study Design	Area of Treatment	Number of Patients	Main Results of Cal/BDP versus Comparator
Cal/BDP ointment BID	BDP ointment BID, Cal ointment BID	4 weeks	Randomized clinical trial	Body	1106	Greater reduction in PASI [61]
Cal/BDP ointment OD	BDP ointment OD, Cal ointment OD, ointment vehicle OD	4 weeks	Randomized clinical trial	Body	1603	Greater reduction in PASI, increased proportion of patients with absence/very mild disease (IGA) [62]
Cal/BDP ointment BID	BDP ointment BID, Cal ointment BID, ointment vehicle BID	4 weeks	Randomized clinical trial	Body	1043	Greater reduction in PASI [63]
Cal/BDP ointment OD	BDP ointment BID	12 weeks	Randomized clinical trial	Body	66	Greater reduction in PASI [64]
Cal/BDP ointment OD	0.1% Dexamethasone cream OD	4 weeks	Randomized clinical trial	Body	30	Greater reduction in total score of itching, erythema, infiltration, scales [68]
Cal/BDP ointment OD	Betamethasone valerate dressing (0.1%) OD	4 weeks	Randomized clinical trial (non-inferiority)	Body	324	Not inferior regarding TSS-4 [67]
Cal/BDP ointment OD	Clobetasol propionate spray (0.05%) BID	4 weeks	Clinical trial	Body	93	Reduced proportion of patients with clear/almost clear disease (ODS) [66]
Cal/BDP ointment OD	BDP ointment BID, Cal ointment BID	30 days	Clinical trial	Body	30	Greater reduction in mPASI and microvascular restoration [57]
Cal/BDP ointment OD	BDP ointment BID, Cal ointment BID	4 weeks	Pooled analysis	Body	2566	Greater reduction in PASI [82]
Cal/BDP ointment OD	BDP ointment BID, Cal ointment BID	4 weeks	Pooled analysis	Body	3406	Increased proportion of patients with PASI 50 and PASI 75 ^#^ [83]
Cal/BDP ointment OD/BID	BDP ointment OD/BID, Cal ointment OD/BID, ointment vehicle OD/BID, tacalcitol OD	4 weeks	Pooled analysis	Body	6050	Greater reduction in PASI ^#^ [84]
Cal/BDP ointment OD/BID	BDP ointment OD/BID, Cal ointment OD/BID, Cal/clobetasol butyrate (0.05%) ointment OD, Cal/betamethasone valerate ointment (0.1%) OD, placebo ointment OD/BID, tacalcitol (0.0004%) ointment OD	4 weeks	Meta-analysis	Body	6708	Ranked as the most efficacious [85]
Cal/BDP gel OD	BDP gel OD, Cal gel OD, gel vehicle OD	8 weeks	Randomized clinical trial	Scalp	1505	Increased proportion of patients with absent/very mild disease (IGA) [69]
Cal/BDP gel OD	BDP gel OD	8 weeks	Randomized clinical trial	Scalp	218	Greater reduction in TSS (redness, thickness, scaliness) [70]
Cal/BDP gel OD	BDP gel OD, Cal gel OD	8 weeks	Randomized clinical trial	Scalp	1417	Increased proportion of patients with absent/very mild disease (IGA) [71]
Cal/BDP gel OD	Cal gel OD, BDP gel OD, gel vehicle OD	8 weeks	Randomized clinical trial	Body	364	Increased proportion of responders (IGA) [72]
Cal/BDP gel OD	Cal gel OD, gel vehicle OD	8 weeks	Randomized clinical trial	Body	1152	Increased proportion of patients with controlled disease (IGA) [73]
Cal/BDP gel OD	BDP gel OD ^+^, mometasone emulsion (0.1%) OD ^+^	4 weeks ^+^	Real-world data (daily clinical practice)	Scalp	183	Similar results in terms of disease severity and quality of life, although mometasone emulsion showed greater acceptability by patients and physicians in terms of perceived efficacy, tolerability, and compliance [86]
Cal/BDP gel OD	BDP gel OD, Cal gel OD, gel vehicle OD	1 week	Pooled analysis	Scalp	2920	Increased proportion of patients with absent/very mild disease (IGA) [87]
Cal/BDP gel OD	Placebo gel OD/BD, Cal gel OD/BD, Cal/coal tar (4%) shampoo BD, betamethasone valerate gel BD, salicylic acid (0.5%)/coconut oil (1.0%)/coal tar (1.0%) shampoo OD	4 weeks	Meta-analysis	Scalp	6053	Greater response in terms of IGA or TSS [88]
Cal/BDP foam OD	BDP foam OD, Cal foam OD	4 weeks	Randomized clinical trial	Body and scalp	302	Increased proportion of patients with treatment success (PGA) for body psoriasis and no differences for scalp psoriasis [74]
Cal/BDP foam OD	Cal/BDP ointment OD, BDP foam OD, foam vehicle OD	4 weeks	Randomized clinical trial	Body	24	Greater reduction in total clinical score (erythema, scaling, and lesional thickness) [78]
Cal/BDP foam OD	Betamethasone valerate plaster (2.25 mg) OD	4 weeks	Randomized clinical trial	Body	35	Greater reduction in total clinical score (erythema, scaling, and infiltration) [79]
Cal/BDP foam OD	Clobetasol propionate cream (0.05%) OD	4 weeks	Randomized clinical trial	Body	36	Increased proportion of patients with total clinical score ≤1, better patient satisfaction, and greater reduction in hyperkeratosis/acanthosis [60]
Cal/BDP foam (unspecified dosage)	Cal/BDP ointment, BDP foam, Cal foam, foam vehicle, ointment vehicle (unspecified dosages)	Unspecified dosage, 4 weeks	Pooled analysis	Body	1104	Increased proportion of patients with treatment success (PGA) [89]

* Cal 0.005% and BDP 0.064%. ^†^ For Cal and BDP, same concentration as that in the Cal/BDP combined formulation; ^#^ numerical differences, statistical analysis was not conducted; ^+^ treatments were prescribed for most patients as OD application. Both therapies were administered in accordance with the summary of product characteristics, which recommend that the treatment should be administered for a maximum of 3 weeks for mometasone or 4 weeks for Cal or Cal/BDP. The final decision on the duration of the therapy was made by the physicians. BDP: betamethasone dipropionate, BID: twice daily, Cal: calcipotriol, IGA: Investigator’s Global Assessment, mPASI: Modified Psoriasis Area and Severity Index, OD: once daily, ODS: Overall Disease Severity, PASI: Psoriasis Area Severity Index, PASI 50: 50% improvement of PASI from baseline, PASI 75: 75% improvement of PASI from baseline, PGA: Physician Global Assessment, TSS: total severity score, TSS-4: total severity score of four items.
